# Optical Sensitivity of Camera-Like Eyes to White Light

**DOI:** 10.3390/vision5040044

**Published:** 2021-09-28

**Authors:** Irina P. Shepeleva

**Affiliations:** Laboratory of Visual Physiology, Pavlov Institute of Physiology, Russian Academy of Sciences, Makarova emb. 6, 199034 St. Petersburg, Russia; i.p.shepeleva@yandex.ru

**Keywords:** optical sensitivity, camera-like eye, gastropod mollusks, humans

## Abstract

Gastropod mollusks are convenient model organisms for studying the functioning of the visual system. The purpose of this work is to estimate the value of the optical sensitivity to white light for the camera-like eyes of gastropod mollusks and humans and analyze its effect on the spatial resolving power in two regions of the retina: in the center—for single photoreceptors of the first/second type in a mollusk and single cones in humans—and in the periphery—for single photoreceptors of the first/second type in a mollusk, as well as for single rods/cones and their groups, subject to spatial summation in humans. The methods of histology, light and transmission electron microscopy, morphometry, calculations and methods of statistical analysis are used in the work. In a mollusk, with a fixed pupil area, the value of the optical sensitivity of the eye to white light in the center of the retina for single photoreceptors of the first/second type is 0.5/0.006 μm^2^·sr and in the periphery of the retina, 0.9/0.009 μm^2^·sr. In humans, at the minimum and maximum pupil area, respectively, the value of the optical sensitivity of the eye to white light in the center of the retina (foveola) for single cones varies from 0.00053 to 0.028 μm^2^·sr, and in the periphery of the retina (far periphery) for single rods from 0.011 to 0.575 μm^2^·sr, for single cones from 0.025 to 1.319 μm^2^·sr, for the groups of rods from 3859 to 204,094 μm^2^·sr and for the groups of cones from 2.5 to 131 μm^2^·sr. The value of the optical sensitivity of the eyes to white light for single photoreceptors of the first/second type in both regions of the retina in a mollusk, as well as for single cones in the center and groups of rods/cones in the periphery of the retina in humans, corresponds to the ambient light conditions during periods of activity and does not affect the spatial resolving power.

## 1. Introduction

White light in contrast to monochromatic light is the main physical stimulus for vision in nature [1,2]. However, vision, which is characterized by the spatial resolving power of the eye, is impossible unless the eye collects a sufficient amount of light, namely, has an adequate optical sensitivity to ambient light [3,4,5]. This requirement is generally met without difficulty in a terrestrial environment in high light conditions [5]. Representatives of some groups of invertebrates, including gastropod mollusks—convenient model organisms for studying the functioning of the visual system—, and some groups of vertebrates, including humans, live in such conditions. Both those and others see the world around them by the help of camera-like eyes [6,7,8,9,10,11,12,13]. The eyes of gastropod mollusks and humans consist of a different number of components, but contain five identical basic components necessary for the formation of a camera-like eye: the outer shell, represented by the cornea and the eye capsule/sclera; the inner shell, represented by the retina; the pupil; the lens; the vitreous body [14]. The purpose of this work is to estimate the value of the optical sensitivity to white light and analyze its effect on the spatial resolving power of the camera-like eyes of gastropod mollusks and humans. A terrestrial mollusk (a snail) was chosen, with eyes adapted to vision in daytime light at a high level of illumination, similarly to the human eye [15,16].

## 2. Materials and Methods

### 2.1. Animals

Research was conducted on adults of *Helicigona lapicida* (Linnaeus, 1758), a terrestrial pulmonate gastropod mollusk. The mollusks were collected on the island of Öland (Sweden) in June 2004 and kept in a terrarium with soil at room temperature and natural light, fed with dandelion leaves.

### 2.2. Histology and Microscopy

Eye tentacles of mollusks adapted to darkness were used for light and transmission electron microscopy. The tentacles were sequentially fixed in a 2.5% solution of glutaraldehyde in 0.1 M cacodylate buffer (pH = 8.0) for 2 h at 4 °C and in a 1% solution of osmium tetroxide in the same buffer for 1 h at 4 °C. The material was dehydrated in a series of alcohols of increasing concentration, impregnated with absolute acetone, as well as with a mixture of acetone and resin, and filled in with resin. Semithin (2 μm) and ultrathin (70 nm) sections were cut using an ultramicrotome V LKB 2088 (Leitz, Oberkochen, Germany). Semithin sections were stained with a 0.5% solution of toluidine blue with the addition of 1% sodium carbonate and placed under a cover glass. Sections were examined using a Zeiss Axiophot light microscope (Carl Zeiss, Oberkochen, Germany) and photographed using an Olympus DP 50 digital camera (Olympus, Tokyo, Japan). Ultrathin sections were sequentially contrasted with 2% solution of uranium acetate and 0.1% solution of lead citrate. Sections were examined using a JEOL JEM–1230 transmission electron microscope (JEOL, Tokyo, Japan) and photographed using an AMT–X100 digital camera.

### 2.3. Morphometry

Parameter measurements were performed on photographs of eye sections.

### 2.4. Statistical Analysis

The average values of the obtained data and their standard deviations were calculated [17].

## 3. Results and Discussion

### 3.1. Spatial Resolving Power and Optical Sensitivity of a Camera-like Eye

The spatial resolving power of an eye is the ability to perceive two points separately with a minimum angular distance between them [18]. It depends on the diameter of the photoreceptor cells, the density of their arrangement and the focal length of the optical system. Increasing the spatial resolving power of an eye is achieved by reducing the diameter of the photoreceptor cells to a minimum (up to about 1 μm), simultaneously increasing the density of their arrangement and/or lengthening the focal length [3,19]. The spatial resolving power of an eye (*R*, rad^−1^) for a hexagonal arrangement of photoreceptor cells was calculated by using Formula (1) [3,20]:

(1) $$R=\frac{f}{\surd 3p}$$

where *f* is the focal length of the optical system of the eye and *p* is the distance between the centers of neighboring photoreceptor cells.

The primary condition for the realization of the spatial resolving power of the eye is an adequate optical sensitivity of the eye to available light [3,4,19]. This is because the intensity of a light stimulus is measured by photoreceptor cells as the average rate of the arrival of photons. If only a few photons arrive over a long period of time, then the photoreceptor cells cannot accurately determine the intensity of the stimulus and its location in space. In order to cope with these tasks, and to detect small differences in the intensity of stimuli and discern more details, the photoreceptor cells need to absorb as many photons as possible. The latter depends on the amount of available light in the environment and the capabilities of the eye [4].

The optical sensitivity of an eye is the ratio of the number of photons absorbed by a photoreceptor cell to the number of photons emitted per steradian of a solid angle from a unit area of an extended light source. The optical sensitivity of an eye to white light (*S_w_*, μm^2^·sr) was calculated using Formula (2):

(2) $$S_{w\;=\;}{\left(\begin{array}{c} \pi \\ 4 \end{array}\right)}^{2}A^{2\;}{\left(\begin{array}{c} d\\ f \end{array}\right)}^{2}F$$

where *A* is the diameter of the pupil, *d* is the cross-sectional diameter of the light-sensitive part of the photoreceptor cell, *f* is the focal length of the optical system of the eye and *F* is the total fraction of the incident light absorbed by the photoreceptor cell. The total absorbed fraction of the incident light (*F*) was calculated using Formula (3):

(3) $$F=\frac{kl}{(2.3+kl)}$$

where *k* is the absorption coefficient of the visual pigment, which characterizes the fraction of the incident light absorbed by each unit (1 μm) of the length of the light-sensitive part of the photoreceptor cell [21], and *l* is the length of the light-sensitive part of the photoreceptor cell.

The given formula, Formula (2), has been confirmed experimentally on invertebrates [22]. It should be noted that in the case of any intervention in human eyes, it is necessary to use disinfectants correctly [23,24]. Values of the optical sensitivity of the eyes obtained by this formula have been generally used for a comparative analysis in invertebrates and vertebrates [2,3]. Formulas (2) and (3) show that the optical sensitivity of an eye is affected by several parameters: the area of the pupil (*πA*^2^/4, µm^2^), which depends on its diameter; the cross-sectional area of the light-sensitive parts of photoreceptor cells (*πd*^2^/4*f*^2^, sr), which depends on their cross-sectional diameter and determines the size of the viewing solid angle of visual space; the total absorbed fraction of the incident light (*F*), which depends on the absorption coefficient of the visual pigment and the length of the light-sensitive parts of photoreceptor cells. Thus, the optical sensitivity to white light is determined by the morphological and optical properties of the eye together with the morphological and absorption properties of photoreceptor cells of the retina [2,21].

In mollusks and humans, the optic part of the retina is divided into central and peripheral regions. Both regions are homogeneous in the mollusk and are subdivided into zones in the human [14,25]. Thus, in humans, the central region of the retina, or macula, contains the fovea (central pit), in which the foveola (bottom) with the center (umbo), the declivity and the thick margin are distinguished, and which is surrounded by the parafovea and perifovea. In the peripheral region of the retina, with the distance from the central region, the near, middle, far and extreme periphery are distinguished [25]. The central and peripheral regions of the retina differ in mollusks and humans by the density of the arrangement of photoreceptor cells and, respectively, in the spatial resolving power of the eye. In humans, they can also differ in cellular composition [14,25,26,27,28,29]. Thus, in the mollusk, the spatial resolving power of the eye is higher in the center of the retina and lower in its periphery. Each region contains one kind of photoreceptor cells of the first and second type [14] (Table 1 and Table 2). In humans, the foveola of the central region of the retina is the zone with a maximum spatial resolving power. The latter does not contain rods and short-wave cones, but is filled only with middle-wave and long-wave cones. In other zones of the central region of the retina, as well as in all zones of the peripheral region with a lower spatial resolving power, photoreceptor cells are represented by one kind of rods and three kinds of cones [25,26,27] (Table 3 and Table 4). Therefore, this work estimated the value of the optical sensitivity to white light and analyzed its effect on the spatial resolving power of the camera-like eyes for single photoreceptor cells of the first/second type in the center and in the periphery of the retina in a mollusk and for single cones in the foveola, as well as for single rods/cones and their groups, subject to spatial summation in the far periphery of the retina in humans.

### 3.2. The Influence of the Optical Sensitivity to White Light on the Spatial Resolving Power of the Camera-like Eyes of Mollusks and Humans

#### 3.2.1. Mollusk

In the mollusk, at constant parameters of the eye—the diameter (area) of the pupil, the length of the focal length of the optical system and the absorption coefficient of the visual pigment of single photoreceptor cells of both types, the variable parameters of the latter—, the cross-sectional diameter and the length of the light-sensitive parts make different contributions to the value of the optical sensitivity of the eye depending on the region of the retina and the type of cells (Table 1 and Table 2). Accordingly, in the center of the retina, the cross-sectional diameter of the light-sensitive parts of single photoreceptor cells of the first and second type was less than their length; therefore, it contributed less to the value of the optical sensitivity of the eye, whereas in the periphery of the retina, it was vice versa. The differences between the cross-sectional diameter and the length of the light-sensitive parts of single photoreceptor cells of each type was less pronounced in the center and more in the periphery of the retina, and, probably, because of the size characteristic for each type of photoreceptor cells, they were more evident in the cells of the first type than the second, both in the center of the retina and its periphery. All these differences were due to an increase in the cross-sectional diameter and a simultaneous decrease in the length of the light-sensitive parts of single photoreceptor cells of the first and second types in the periphery of the retina compared with the center, which led to a decrease in the total absorbed fraction of the incident light and an increase in the optical sensitivity of the eye in this region by 1.8 and 1.5 times, respectively. Optical sensitivity improved because the increase in the cross-sectional diameter of the light-sensitive parts of single photoreceptor cells exceeded the decrease in their length. Among the last four listed parameters of single photoreceptor cells of both types—the cross-sectional diameter and the length of the light-sensitive parts, the total absorbed fraction of the incident light and the optical sensitivity—between the center and the periphery of the retina, the smallest differences were found in the length of the light-sensitive parts and, accordingly, in the total absorbed fraction of the incident light and the greatest in the optical sensitivity. Changing each of these parameters manifested to a greater extent in single photoreceptor cells of the first type than the second. A comparison of the parameters of single photoreceptor cells of the first type to the parameters of single photoreceptor cells of the second type in each region of the retina showed that, in the center, the smallest differences were characteristic for the cross-sectional diameter of the light-sensitive parts and the greatest—for the optical sensitivity (83 times), and in the periphery—for the length of the light-sensitive parts/the total absorbed fraction of the incident light and the optical sensitivity (100 times), respectively. Significant differences in the value of the optical sensitivity between single photoreceptor cells of the first and second type in the center of the retina were possible in view of significant differences in the length and the cross-sectional diameter of the light-sensitive parts, whereas in the periphery of the retina—on the contrary—in the cross-sectional diameter and the length of the light-sensitive parts. Thus, in the mollusk, the optical sensitivity of the eye for single photoreceptor cells of both types was lower in the center of the retina and higher in its periphery, and, also, for single photoreceptor cells of the first type was higher than for single photoreceptor cells of the second type in both regions of the retina.

It is known that organisms with daytime activity tend to have an optical sensitivity of eyes to white light below one, twilight activity from 1 to 100, and nighttime activity of more than 100 [19]. In the mollusk, the value of the optical sensitivity of the eye for single photoreceptor cells of the first and second type varied from 0.5 and 0.006 μm^2^·sr in the center of the retina to 0.9 and 0.009 μm^2^·sr in the periphery of the retina and corresponded to daylight conditions (Table 1). The motor activity of the mollusk living on an open rocky seashore is diurnal [15,16]. Obviously, the value of the optical sensitivity of the eye for single photoreceptor cells of both types correlates with the habitat light conditions during periods of motor activity. In addition, the mollusk apparently has a spatial summation of signals from photoreceptor cells both in the center and periphery of the retina [13]. However, its contribution to increasing the optical sensitivity of the eye remains unclear. Also, the value of the optical sensitivity of the eye for single photoreceptor cells of the first and second type differed between the center and the periphery of the retina by 1.8 and 1.5 times, and between single photoreceptor cells of the first and second type in the center and in the periphery by 83 and 100 times, respectively (Table 2). These differences reflect the ability of single photoreceptor cells of the first and second type in the periphery of the retina compared with the center, as well as single photoreceptor cells of the first type compared with single photoreceptor cells of the second type in the center and in the periphery of the retina to absorb a greater number of photons and function at a lower (by 1.8 and 1.5 times and 83/100 times, respectively) level of illumination, which can occur under different weather conditions during the day. Thus, the optical sensitivity of the mollusk eye to white light did not affect the spatial resolving power in the center and in the periphery of the retina.

#### 3.2.2. Human

In humans, at accommodation rest, the only single constant parameter is the length of the focal length of the optical system. Not only do the cross-sectional diameter and length of the outer segments of rods and cones become variable parameters, but, depending on the lighting conditions, the diameter of the pupil changes reflexively,—from 1.1 to 8.0 mm [36], i.e., 7.3 times—, and, accordingly, its area—from 0.95 to 50.24 mm^2^, i.e., 52.88 times. In addition, differences were found in the absorption coefficient of the visual pigments of rods and cones. All these variable parameters contribute differently to the value of the optical sensitivity of the eye, depending on the region of the retina and the type of cells (Table 3 and Table 4). Thus, in the center of the retina (foveola), there were no rods, and in the far periphery of the retina in single rods, the cross-sectional diameter of the outer segments was less than their length, thereby contributing less to the value of the optical sensitivity of the eye. The latter increased in direct proportion to the pupil area, reaching the corresponding maximum difference of 52.27 times. In the center and in the periphery of the retina, in single cones, just as in single rods, the cross-sectional diameter of the outer segments was less than their length; therefore, making a smaller contribution to the value of the optical sensitivity of the eye, which increased in direct proportion to the pupil area by 52.83 and 52.76 times, respectively. However, in the periphery of the retina in single cones, this contribution was more significant than in the center of the retina in single cones and in the periphery of the retina in single rods. This was due to an increase in the cross-sectional diameter and a simultaneous decrease in the length of the outer segments of single cones in the periphery of the retina, as compared to the center, which led to a decrease in the total absorbed fraction of the incident light and an increase in the optical sensitivity of the eye in this region by 47 times both at the minimum and maximum pupil area. The increase in the optical sensitivity resulted from the fact that the increase in the cross-sectional diameter of the outer segments of single cones exceeded the decrease in their length. Among the last four listed parameters of single cones—the cross-sectional diameter and the length of the outer segments, the total absorbed fraction of the incident light and the optical sensitivity—between the center and the periphery of the retina, the smallest differences were found in the total absorbed fraction of the incident light and the greatest in the optical sensitivity. The lowest value of the optical sensitivity of the eye was observed in single cones in the center of the retina at the minimum pupil area and the highest in single cones in the periphery of the retina at the maximum pupil area. Also, for single cones, the value of the optical sensitivity of the eye at the maximum pupil area in the center of the retina was comparable with the value of the optical sensitivity of the eye at the minimum pupil area in the periphery of the retina. This meant that the ability of single cones to absorb photons in the center of the retina was comparable to that in the periphery of the retina, at least for the maximum and minimum pupil area, respectively. A comparison of the parameters of single rods with the parameters of single cones in the periphery of the retina showed that the smallest differences were characteristic for the absorption coefficient of the visual pigments of the cells and the greatest for the optical sensitivity (by 2.3 times both at the minimum and maximum pupil area). The differences in the value of the optical sensitivity of the eye between single rods and single cones in favor of the latter in the periphery of the retina for any pupil area were possible mainly due to the difference in the cross-sectional diameter of their outer segments, since the differences in the absorption coefficient of the visual pigments and the length of the outer segments were mutually compensated and led to a similar value of the total absorbed fraction of the incident light. Thus, in humans, the value of the optical sensitivity of the eye for single cones was lower in the center of the retina and higher in its periphery, and also for single rods was lower than for single cones in the periphery of the retina.

In humans, the value of the optical sensitivity of the eye for single rods in the periphery of the retina varied from 0.011 μm^2^·sr at a minimum pupil diameter (area) of 1.1 mm (0.95 mm^2^) to 0.575 μm^2^·sr at a maximum pupil diameter (area) of 8.0 mm (50.24 mm^2^), while for single cones in the center from 0.00053 to 0.028 μm^2^·sr and in the periphery from 0.025 to 1.319 μm^2^·sr, respectively (Table 3). The value of the optical sensitivity of the eye for single rods in the periphery and for single cones in the center of the retina at any pupil diameter (area), as well as for single cones in the periphery of the retina at pupil diameter (area) less than 6.5 mm (33.17 mm^2^) (*S_w_* = 0.9 μm^2^·sr), did not exceed one and corresponded to the conditions of daylight. At a pupil diameter greater than 6.5 mm, the value of the optical sensitivity of the eye for single cones in the periphery of the retina exceeded one and already corresponded to the conditions of twilight lighting. The calculated values of the optical sensitivity of the eye were unambiguous only for single cones in the center of the retina, where spatial summation of signals was absent and increasing the sensitivity by the help of signal amplification is impossible [27]. Therefore, single cones in the center of the retina can only function in daylight. The value of the optical sensitivity of the eye for single cones in the periphery of the retina was 47 times higher than for single cones in the center of the retina, and for single cones was 2.3 times higher than for single rods in the periphery of the retina by both at the minimum and maximum pupil area (Table 4). These differences reflected the ability of single cones in the periphery compared with single cones in the center of the retina, as well as single cones compared with single rods in the periphery of the retina to absorb more photons and function at a lower (by 47 and 2.3 times, respectively) illumination level. However, unlike the center, the periphery of the retina is characterized by such convergence of rods and cones to ganglion cells, in which each ganglion cell can summarize signals from 600 rods and 10 cones [38,39]. Therefore, in the periphery of the retina, the calculated value of the optical sensitivity of the eye for single rods, which was lower than one, increased significantly for the group of rods—by 350,818 и 354,946 times at the minimum and maximum pupil area, respectively—due to a much stronger convergence and, accordingly, summation of signals than in cones (Table 3 and Table 4). Consequently, due to spatial summation, for any pupil diameter (area), rods in the far periphery of the retina can function in twilight and night illumination. Compared to single rods in the periphery of the retina, the calculated value of the optical sensitivity of the eye for single cones, which was lower and slightly higher than one, for the group of cones increased less significantly—by 100 and 99 times at the minimum and maximum pupil area, respectively (Table 3 and Table 4). Therefore, for any pupil diameter (area), cones in the far periphery of the retina can function in twilight lighting. Spatial summation led to the fact that the value of the optical sensitivity of the eye for the group of cones in the periphery of the retina exceeded that for single cones in the center of the retina by 4717 and 4679 times, and the value of the optical sensitivity of the eye for the group of rods exceeded that for the group of cones in the periphery of the retina by 1544 and 1558 times at the minimum and maximum pupil area, respectively (Table 3 and Table 4). Also, now in the periphery of the retina in groups of rods and cones, the cross-sectional diameter, rather than the length of their outer segments, contributed more to the value of the optical sensitivity of the eye, which increased in direct proportion to the pupil area, reaching the corresponding maximum difference of 52.89 and 52.40 times. In addition, the highest value of the optical sensitivity of the eye was observed not in single cones in the periphery of the retina at the maximum pupil area, but in the group of rods in the periphery of the retina at the maximum pupil area. All values of the optical sensitivity of the eye obtained for single cones in the center of the retina (foveola), as well as the values possible due to spatial summation for the groups of rods and cones in the far periphery of the retina, were consistent with the literature data, according to which cones function in daylight and twilight lighting, and rods—in night and twilight and with less efficiency—in daylight lighting [27,40,41]. It was obvious that the value of the optical sensitivity of the eye for single cones in the center of the retina (foveola), as well as for the groups of rods and cones in the periphery (far periphery) of the retina, was in accordance with the light conditions of the human environment during periods of activity. Thus, the optical sensitivity of the human eye to white light did not affect the spatial resolving power in the center (foveola) and in the periphery (far periphery) of the retina.

## 4. Conclusions

The estimation of the value of the optical sensitivity to white light and the analysis of its effect on the spatial resolving power in the center and in the periphery of the retina for the camera-like eyes of gastropod mollusks and humans, allowed to make the following conclusions. Among the five parameters considered for the estimation of the value of the optical sensitivity of the eyes, two parameters were variable in the mollusk—the cross-sectional diameter and the length of the light-sensitive parts of single photoreceptor cells of both types—and four parameters in humans—the diameter (area) of the pupil, the cross-sectional diameter and the length of the outer segments of single rods and cones and, also, the absorption coefficient of their visual pigments. All of them contributed differently to the value of the optical sensitivity of the eyes, depending on the region of the retina and the type of cells. In the mollusk, the length of the light-sensitive parts of single photoreceptor cells of the first and second type contributed more to the value of the optical sensitivity of the eye in the center of the retina, and the cross-sectional diameter in the periphery of the retina. In humans, in the periphery of the retina in single rods as well as in the center and periphery of the retina in single cones, the length of their outer segments provided a greater contribution to the value of the optical sensitivity of the eye, which increased in direct proportion to the pupil area. In the periphery of the retina, in comparison with the center, the value of the optical sensitivity of the eye in the mollusk for single photoreceptor cells of the first and second type was 1.8 и 1.5 times higher, respectively; in humans for single cones, it was 47 times higher. In both mollusks and in humans, such an increase was due to the fact that the increase in the cross-sectional diameter of the light-sensitive parts of single photoreceptor cells of both types and the outer segments of single cones, respectively, exceeded the decrease in their length. The value of the optical sensitivity of the eye in the mollusk for single photoreceptor cells of the first type was higher than for single photoreceptor cells of the second type by 83 times in the center and by 100 times in the periphery of the retina, while in humans for single rods was lower than for single cones by 2.3 times in the periphery of the retina. In the mollusk, significant differences in the value of the optical sensitivity between single photoreceptor cells of the first and second type in the center of the retina were possible in view of the significant differences in the length of the light-sensitive parts and their cross-sectional diameter; in the periphery of the retina it was vice versa. In humans, insignificant differences in the value of the optical sensitivity between single rods and cones in the periphery of the retina were mainly caused by the difference in the cross-sectional diameter of their outer segments. The calculated values of the optical sensitivity of the eye for single photoreceptor cells of the first and second type in the mollusk in the center and in the periphery of the retina, as well as for single rods and cones in humans in the periphery of the retina, were not unambiguous and increased under the influence of spatial summation. The latter has not been studied in the mollusk, but in humans it contributed to the fact that the value of the optical sensitivity of the eye for the group of cones in the periphery of the retina exceeded that for single cones in the center of the retina by 4717 and 4679 times, and the value of the optical sensitivity of the eye for the group of rods exceeded that for the group of cones in the periphery of the retina by 1544 and 1558 times at the minimum and maximum pupil area, respectively. In humans, significant differences in the value of the optical sensitivity between the groups of rods and cones in the periphery of the retina were mainly caused by the difference in the cross-sectional diameter of their outer segments. In general, the calculated value of the optical sensitivity of the eyes to white light in the center and in the periphery of the retina for single photoreceptor cells of the first/second type in the mollusk, as well as for single cones in the center and the groups of rods/cones in the periphery of the retina in humans, corresponded to the ambient light conditions during periods of activity and, thus, did not affect the spatial resolving power. It should be noted that the optical sensitivity of an eye to white light largely depends on the properties of the photoreceptor cells of the retina. Therefore, in mollusks and humans, the calculated value of the optical sensitivity of the eyes to white light did not affect the spatial resolving power at a cellular level. This is how the optical sensitivity of an eye to white light differs from the principle optical disadvantage—the diffraction of light at the pupil of an eye, which depends on the diameter of the pupil. Therefore, the diffraction of light at the pupil of an eye determines the spatial resolving power at an eye level [3,4,19].

## Figures and Tables

**Table 1:** Parameters of the mollusk eye.

Parameter	Retinal Region	Mollusk	References
Size of the eye (anteroposterior × horizontal axis), μm	-	189 × 228	[15]
Focal length of the optical system of the eye *f*, μm	-	162	[15]
Distance between the centers of neighboring single photoreceptor cells of the first/second type *p*, μm	center	11/6.0	[15]
	periphery	19 ± 0.5 */11 ± 0.2 * (*n* = 10/5)	-
Spatial resolving power of the eye for single photoreceptor cells of the first/second type *R*, rad^−1^	center	8.5/15.8	[15]
	periphery	4.9 */8.5 *	
Diameter of the pupil *A*, μm	-	103	[15]
Area of the pupil *S*, μm^2^	-	8328	
Cross-sectional diameter of the light-sensitive part of a single photoreceptor cell of the first/second type *d*, μm	center	8.0/2.0	[15]
	periphery	12.8 ± 0.3 */2.5 ± 0.09 * (*n* = 10/6)	-
Absorption coefficient of the visual pigment of a single photoreceptor cell of the first/second type *k*, μm^−1^	-	0.0067/0.0067	[30]
Length of the light-sensitive part of a single photoreceptor cell of the first/second type *l*, μm	center	11/2.2	[15]
	periphery	8.0 ± 0.09 */2.1 ± 0.13 * (*n* = 10/6)	-
Total fraction of the incident light absorbed by the light-sensitive part of a single photoreceptor cell of the first/second type *F*	center	0.031/0.0064	-
	periphery	0.023 */0.0061 *	-
Optical sensitivity of the eye to white light for a single photoreceptor cell of the first/second type *S_w_*, μm^2^·sr	center	0.5/0.006	[15]
	periphery	0.9 */0.009 *	-

Note: When calculating the optical sensitivity of the eye to white light, light losses due to reflection, scattering and absorption by the ocular media were not taken into account [2]. Parameters that were not referenced and were not marked with an asterisk were calculated by the author on the basis of data from the specified works. The parameters indicated by an asterisk were obtained by the author from the sections of mollusks’ eyes or calculated by the author on the basis of these data and data from the specified works.

**Table 2:** The ratio of the parameters of the mollusk eye.

The Ratio of the Parameters	Retinal Region	Mollusk
Diameter and length of the light-sensitive part of a single photoreceptor cell of the first/second type	center	0.7/0.9
	periphery	1.6/1.2
Cross-sectional diameter of the light-sensitive part of a single photoreceptor cell of the first/second type in the center and in the periphery	-	0.6/0.8
Length of the light-sensitive part of a single photoreceptor cell of the first/second type in the center and in the periphery	-	1.4/1.1
Total fraction of the incident light absorbed by the light-sensitive part of a single photoreceptor cell of the first/second type in the center and in the periphery	-	1.4/1.1
Optical sensitivity of the eye to white light for a single photoreceptor cell of the first/second type in the center and in the periphery	-	0.6/0.7
Cross-sectional diameter of the light-sensitive part of a single photoreceptor cell of the first and second type	center	4.0
	periphery	5.1
Length of the light-sensitive part of a single photoreceptor cell of the first and second type	center	5.0
	periphery	3.8
Total fraction of the incident light absorbed by the light-sensitive part of a single photoreceptor cell of the first and second type	center	4.8
	periphery	3.8
Optical sensitivity of the eye to white light for a single photoreceptor cell of the first and second type	center	83
	periphery	100

**Table 3:** Parameters of the human eye.

Parameter	Retinal Region	Human	References
Size of the eye (anteroposterior × horizontal axis), mm		22.0–24.8 × 24.2	[31]
Focal length of the optical system of the eye *f*, mm		22.3	[32]
Distance between the centers of neighboring single rods/cones *p*, μm	center	–/1.5	[33]
	periphery	6.0/20	[34,35]
Spatial resolving power of the eye for single rods/cones *R*, rad^−1^	center	–/8577	
	periphery	2144/645	
Diameter of the pupil *A*, mm		1.1–8.0	[36]
Area of the pupil *S*, mm^2^		0.95–50.24	
Cross-sectional diameter of the outer segment of a single rod/cone *d*, μm	Center	–/1.0	[33]
	periphery	5.5/10	[26]
Absorption coefficient of the visual pigment of a single rod/cone, *k* μm^−1^		0.028/0.035	[2]
Length of the outer segment of a single rod/cone *l*, μm	Center	–/35	[33]
	periphery	25/13	[37]
Total fraction of the incident light absorbed by the outer segment of a single rod/cone *F*	Center	–/0.348	
	periphery	0.233/0.165	
Optical sensitivity of the eye to white light for a single rod/cone *S_w_*, μm^2^·sr (at *A* = 1.1–8.0 mm)	Center	–/0.00053–0.028	
	periphery	0.011–0.575/0.025–1.319	
Cross-sectional diameter of the outer segments of the group of 600 rods/10 cones *d*, μm	periphery	3300/100	
Optical sensitivity of the eye to white light for the group of 600 rods/10 cones *S_w_*, μm^2^·sr (at *A* = 1.1–8.0 mm)	periphery	3859–204,094/2.5–131	

Note: When calculating the optical sensitivity of the eye to white light, light losses due to reflection, scattering and absorption by the ocular media were not taken into account [2]. Parameters that were not referenced were calculated by the author on the basis of data from the specified works. The distance between the centers of the outer segments of neighboring single rods was calculated by the author from the density of their arrangement from [35] using the formula from [20]. For three kinds of cones, the same cross-sectional diameter and length of the outer segment are given, since they are comparable.

**Table 4:** The ratio of the parameters of the human eye.

The Ratio of the Parameters	Retinal Region	Human
Minimum and maximum diameter of the pupil	-	0.14
Minimum and maximum area of the pupil	-	0.02
Absorption coefficient of the visual pigment of a single rod/cone	-	0.8
Cross-sectional diameter and length of the outer segment of a single rod/cone	center	–/0.03
	periphery	0.2/0.8
Optical sensitivity of the eye to white light for a single rod/cone (at *A* = 1.1–8.0 mm)	center	–/0.02
	periphery	0.02/0.02
Cross-sectional diameter of the outer segment of a single cone in the center and in the periphery	-	0.1
Length of the outer segment of a single cone in the center and in the periphery	-	2.7
Total fraction of the incident light absorbed by the outer segment of a single cone in the center and in the periphery	-	2.1
Optical sensitivity of the eye to white light for a single cone in the center and in the periphery (at *A* = 1.1–8.0 mm)	-	0.02–0.02
Cross-sectional diameter of the outer segment of a single rod and cone in the periphery	-	0.55
Length of the outer segment of a single rod and cone in the periphery	-	1.9
Total fraction of the incident light absorbed by the outer segment of a single rod and cone in the periphery	-	1.4
Optical sensitivity of the eye to white light for a single rod and cone in the periphery (at *A* = 1.1–8.0 mm)	-	0.44–0.44
Optical sensitivity of the eye to white light for a single rod and the group of 600 rods in the periphery (at *A* = 1.1–8.0 mm)	-	0.0000028–0.0000028
Optical sensitivity of the eye to white light for a single cone and the group of 10 cones in the periphery (at *A* = 1.1–8.0 mm)	-	0.01–0.01
Optical sensitivity of the eye to white light for a single cone in the center and the group of 10 cones in the periphery (at *A* = 1.1–8.0 mm)	-	0.0002–0.0002
Optical sensitivity of the eye to white light for the group of 600 rods and the group of 10 cones in the periphery (at *A* = 1.1–8.0 mm)	-	1544–1558

## Data Availability

The data presented in this study are available on request from the corresponding author.
